# Cooperative secretions facilitate host range expansion in bacteria

**DOI:** 10.1038/ncomms5594

**Published:** 2014-08-05

**Authors:** Luke McNally, Mafalda Viana, Sam P. Brown

**Affiliations:** 1 Centre for Immunity, Infection and Evolution, School of Biological Sciences, University of Edinburgh, Ashworth Laboratories, West Mains Road, Edinburgh EH9 3JT, UK; 2 Institute of Evolutionary Biology, School of Biological Sciences, University of Edinburgh, Ashworth Laboratories, West Mains Road, Edinburgh EH9 3JT, UK; 3 Institute of Biodiversity, Animal Health and Comparative Medicine, Graham Kerr Building, University of Glasgow, Glasgow G12 8QQ, UK

## Abstract

The majority of emergent human pathogens are zoonotic in origin, that is, they can transmit to humans from other animals. Understanding the factors underlying the evolution of pathogen host range is therefore of critical importance in protecting human health. There are two main evolutionary routes to generalism: organisms can tolerate multiple environments or they can modify their environments to forms to which they are adapted. Here we use a combination of theory and a phylogenetic comparative analysis of 191 pathogenic bacterial species to show that bacteria use cooperative secretions that modify their environment to extend their host range and infect multiple host species. Our results suggest that cooperative secretions are key determinants of host range in bacteria, and that monitoring for the acquisition of secreted proteins by horizontal gene transfer can help predict emerging zoonoses.

## Abstract (web summary)

Understanding the factors determining pathogen host range is critical for human health. Here, the authors show that bacteria use cooperative secretions to modify their environment and to infect multiple host species, which suggests that cooperative secretions are key determinants of host range in bacteria.

## Graphical abstract

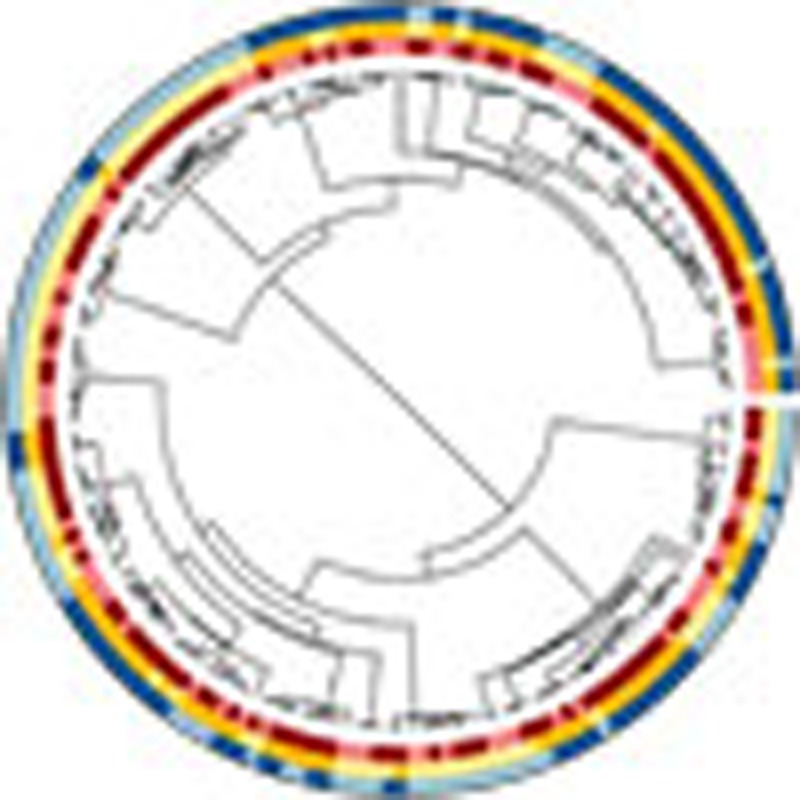

Predicting the emergence of human pathogens is of obvious importance because of both their huge burden on human health and economic cost^1^,^2^,^3^. The majority of these emerging pathogens are zoonotic, that is, they can transmit between humans and animals^4^,^5^. Although some environmental drivers of zoonosis have been identified, such as population density and wildlife biodiversity^5^, the mechanisms by which pathogens extend their host range and become generalists are poorly understood^6^,^7^.

Organisms can achieve generalism by increasing their phenotypic repertoire (for example, by plastically responding to different conditions with different behaviours or the activation of different metabolic pathways), thus becoming tolerant of a wider range of conditions^8^. However, organisms can also achieve generalism by modifying the distinct environments they encounter^9^,^10^ so they resemble a common state to which they are specialized^11^,^12^, in a process often termed ‘environmental modification’^9^. Bacteria modify their environments in many ways, most notably via the secretion of metabolically costly proteins and metabolites, many of which are known to be important virulence factors^13^,^14^. Examples include the secretion of toxins that kill competitors^15^,^16^,^17^, digestive exoenzymes that modify the nutrient environment^18^,^19^ and biofilms that protect bacteria from undesirable environments and/or smother competitors^20^,^21^. By modifying the local environment, these secretions may not only increase the growth of the strains producing them, but also create an environment to which competitors are maladapted. Owing to their extracellular nature, these traits are typically public goods, and have often been studied in terms of their social evolutionary dynamics^13^. However, their role in the evolution of niche breadth remains unexplored. Here we show that these secretions allow pathogenic bacteria to modify and standardize diverse host environments, thus allowing them to expand their host range (Fig. 1).

## Results

### Comparative analysis

How can we distinguish between the strategies of environmental modification via secretions and classical generalism in bacteria? Previous work has suggested that bacteria using a classical generalist strategy will have larger genomes than specialists to deal with multiple distinct environments^22^. For example, classical generalists may evolve additional metabolic pathways to deal with differing nutrient environments. This leads to the prediction that, if classical generalism is the strategy used by bacteria to extend their host range, the ability to infect multiple hosts will be positively correlated with genome size. However, if bacteria use a strategy of modifying host environments via secretions we expect a different genomic signature. First, we predict that, if bacteria use this environmental modification strategy, the ability to infect multiple hosts will be positively correlated with the number of secretions coded in bacterial genomes. The logic for this prediction is that a greater number of secretions coded in the genome will allow bacteria to modify host environments to a greater extent (for example, digestively simplifying nutrient conditions or toxifying the environment for resident competitors). Second, we predict that, if bacteria use this environmental modification strategy, the ability to infect multiple hosts will be negatively correlated with genome size. The logic for this prediction is that investment in modifying and standardizing the external environment leads to a reduction in the requirement for diverse and specific genetic adaptations to multiple distinct environments. We therefore expect that the ability of bacteria to infect multiple host species is positively correlated with secretome size and negatively correlated with genome size if environmental modification via secretions is the major route to host generalism, while we expect it to be positively correlated with genome size if bacteria use a classical generalist strategy.

**Figure 2:**
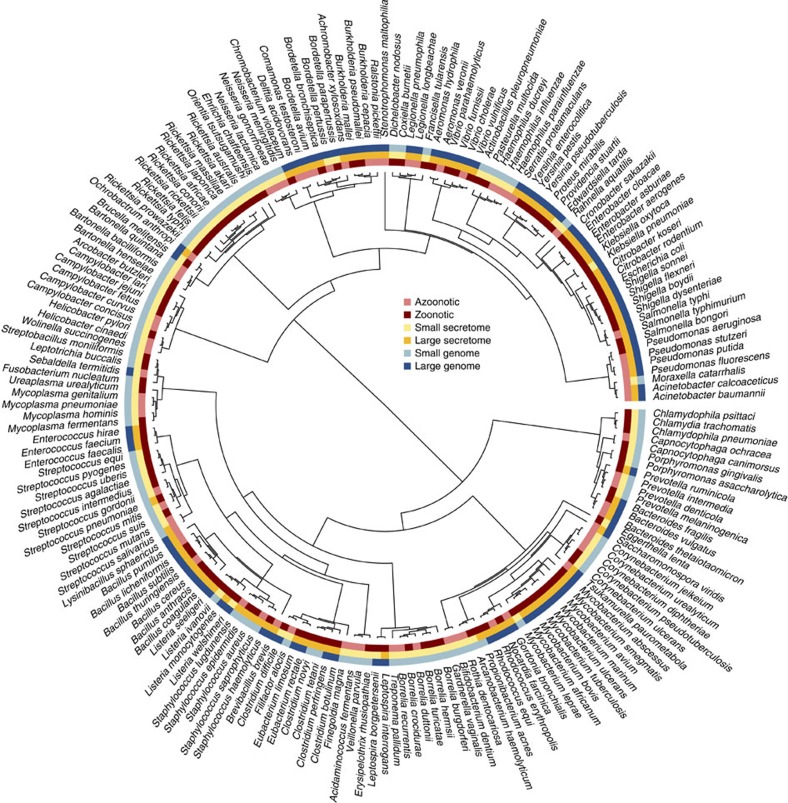
The phylogenetic distribution of zoonosis, genome size and secretome size. The phylogenetic distribution of zoonotic status, secretome size and genome size is shown. Large genomes and secretomes are those greater than the median and small are less than or equal to the median. Note that the tree is ultrametricized for illustrative purposes only.

On the basis of these predictions, we used a phylogenetic comparative analysis^23^ to test whether pathogenic bacteria use environmental modification via secretions to achieve host generalism (Fig. 2 and Supplementary Fig. 1). We gathered data on whether bacteria that infect humans are zoonotic (that is, infect hosts other than humans) from a previous compilation^4^. We also gathered data on bacterial genome sizes and measured investment in secretions by computational prediction of their secretome (that is, the secreted proteome) sizes from the PSORTdb database^24^. The secretome size of bacteria indicates the diversity of secreted proteins that they can use to modify their environment, thus measuring their potential to modify distinct host environments. In total, genome sequences and epidemiological data were available for 191 human pathogen species (121 zoonotic species, 70 azoonotic species). As data for different bacterial species are non-independent owing to their shared evolutionary history, we used the whole-genome-based SUPERFAMILY phylogeny^25^ to account for common ancestry among species. We analysed our data using a Bayesian phylogenetic mixed model (BPMM), with the zoonotic status of each species as a binary response variable, genome and secretome size as predictors, and the phylogenetic relationships among species as a random effect.

**Figure 3:**
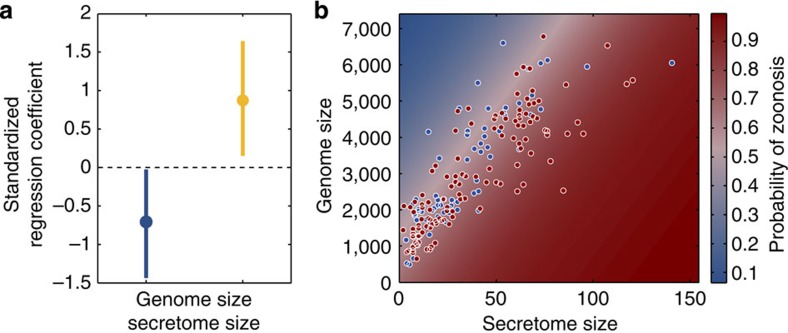
Bacterial secretions increase the ability of pathogens to infect multiple hosts. (**a**) Standardized regression coefficients (multiplied by the standard deviation of the variable) estimated by the BPMM. Dots show the mode of the posterior distributions with lines indicating 95% CIs. Secretome size (yellow) has a positive effect on the probability that a pathogen is zoonotic, whereas genome size (blue) has a negative effect. (**b**) Data and BPMM predictions. Zoonoses are shown in red (*n*=121), whereas specialists are shown in blue (*n*=70). Background colours indicate the predictions of the BPMM.

Consistent with the hypothesis of environmental modification via secretions, we found that larger secretome sizes are associated with a higher probability that a pathogen is zoonotic (Fig. 3, BPMM: parameter estimate (*β*)=3.23 × 10^−2^, 95% credible interval (CI)=5.54 × 10^−3^ to 6.08 × 10^−2^). Also in accordance with the hypothesis of environmental modification via secretions, but counter to the alternative classical generalism model^22^, we found that genome size had a negative effect on the probability that a pathogen is zoonotic (BPMM: *β*=−4.61 × 10^−4^, 95% CI=−1.59 × 10^−5^ to −9.36 × 10^−4^). These results suggest that cooperative environmental modification is the major route to host generalism in pathogenic bacteria.

### Theoretical model

Why do bacteria use environmental modification via secretions to achieve generalism instead of the classical mechanism of increasing their phenotypic repertoire? We now theoretically examine modification of the host environment as a strategy to achieve generalism under a simple nested epidemiological scenario. Our model focuses on the epidemiological consequences of secretions that modify a strain’s environment rather than the conditions for the initial evolution of these secretions, which are already well understood^13^. We use a susceptible–infected–susceptible epidemiological model, with explicit within-host dynamics governed by the replicator equation^26^, to model the dynamics of competing pathogen strains. We consider a scenario where pathogens can potentially infect two different host species, and where transmission between these species is possible. We consider four different strain types: two specialist strains that each infect one of the two host species, classical generalists that can infect both species and environmental modifiers that can infect both species by investment in cooperative modification of the host environments into a common simplified state. We make four key assumptions in our model. First, we assume that a generalist’s growth rate *g* is lower than the growth rate *s* of a specialist within its preferred host (*g*<*s*), that is, that there is a trade-off in the evolution of classical generalism^8^. We further assume that environmental modifiers have a growth rate *bEM*−*c* in a host, where *b* is the benefit from growing in the modified host environment that they create, *c* (*c*<*b*) is the cost of investment in secretions to modify the host environment and *EM* is the frequency of the environmental modifier strain within the host. The growth rate of environmental modifiers is therefore positively frequency dependent (that is, increasing with *EM*) as modification of the host environment is a collective endeavour. This modification of conditions within the disease site is predicted to reduce the growth rates of specialists and classical generalists during co-infections as it creates an environment to which they are maladapted (for example, by modifying the nutrient environment and/or community composition to a new state in which specialists growth rate will be reduced). We model this effect by setting the growth rates of specialists and classical generalists during co-infection with environmental modifiers as *s*(1−*EM*) and *g*(1−*EM*), respectively. Finally, we assume that environmental modifiers’ growth rate in a single strain infection is lower than that of a specialist in their preferred host species (*b*−*c*<*s*).

**Figure 4:**
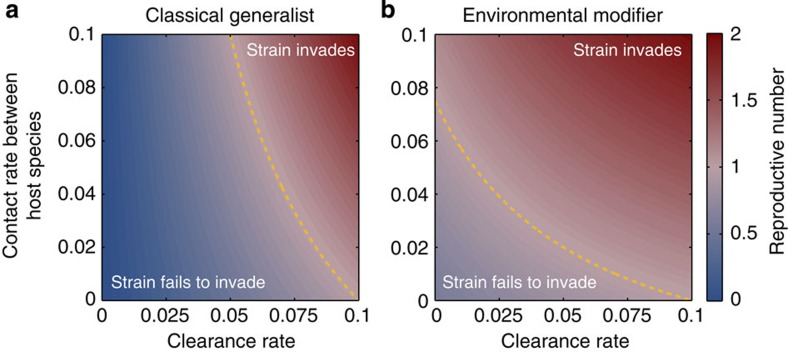
Invading a population of specialists. Plotted is the ‘basic reproductive number’ (number of new infections created per unit time when the pathogen is rare) of classical generalists (**a**) and environmental modifiers (**b**) when invading a population of specialists from our epidemiological model. The *x* and *y* axes are the rate at which infections are cleared (*α*) and the contact rate between host species (*β*_b_), respectively. High reproductive numbers are red and low are blue. The yellow dashed line indicates where the reproductive number equals 1. At values above 1 the strain can invade. Our model predicts that a strain using environmental modification via secretions can invade a resident population of specialist strains under a wider range of conditions than a classical generalist strain can (smaller area above yellow dashed line in **a** than in **b**). Here *s*=1.5, *g*=1, *b*=1.25, *c*=0.25 and the within-host species contact rate, *β*_w_=0.1. Environmental modifiers are better able to invade a population of specialists than classical generalists, despite identical within-host growth rates in single strain infections, as environmental modifiers alter the host environment to a form that specialists are not adapted to. This result holds whenever *b*>*c*.

Our theoretical model shows that strains using a strategy of environmental modification via cooperative secretions can invade populations of specialist pathogens under a wider range of conditions than classical generalists (Fig. 4). Both classical generalists and environmental modifiers are favoured by higher contact rates between host species (favouring generalism) and higher clearance rates of infection (reducing competition with specialists). However, the condition for environmental modifiers to invade the specialist population is less stringent than the condition for classical generalists (that is, their basic reproductive number is always greater). This occurs because of what we refer to as a ‘scorched earth’ effect. Environmental modifiers alter the host environment, increasing their own growth rate, while also reducing the growth rate of any co-infecting specialist, which is not adapted to this modified environment. This means that, even when they have a lower growth rate in single strain infections, environmental modifiers can compete successfully against specialists within a host by sufficiently reducing the specialist’s growth rate relative to their own. We also note that, while we have assumed that modification of the host environment increases the growth rate of environmental modifiers, in principle this same effect may occur when environmental modifiers secretions toxify the environment for themselves also, so long as they reduce the growth rate of specialists to a greater extent^12^.

## Discussion

Our results have major implications for our understanding of the consequences, and evolutionary function, of bacterial sociality. Secretions are generally considered to be social traits in bacteria: they will either help or harm the surrounding cells^13^,^14^. The great abundance of these social secretions has led to a wealth of literature exploring selective forces governing the evolution of bacterial sociality^13^,^27^. Our results show that one of the major consequences of these social traits is niche expansion via environmental modification (whenever environmental modifiers are better adapted to the resulting environmental change), suggesting that elucidating the evolutionary functions of social traits has a key role in understanding microbial ecology and biogeography.

We stress, however, that we are not suggesting that host range expansion is necessarily the adaptive function of secretions in pathogenic bacteria. Bacterial pathogens are often opportunistic, and it has been recognized that many phenotypes of importance in disease may be by-products of selection outside the host environment^28^. Secretions that contribute to environmental modification may have evolved owing to their effects in other environments (for example, soil, vegetation and so on), with the ability to infect new hosts being a by-product or spandrel^29^. While additional hosts colonized via environmental modification may not be of demographic significance for bacteria in all cases, our results suggest that the secretome provides a powerful tool to open up new environments for bacteria to which they can potentially further adapt.

Our results provide strong support for the idea that cooperative secretions are an important driver of host range evolution in bacteria. However, it is possible that some unmeasured ecological or genomic variable that correlates with secretome and genome size in bacteria is the proximate driver of host range expansion. However, our results lead to three key experimentally testable predictions for future work to establish the direct role of environmental modification via cooperative secretions in determining host range. First, we predict that cooperative secretions will simplify and standardize both the nutrient environment and resident bacterial communities across a range of hosts. Second, we predict that strains and/or species with the combination of a large secretome and small genome will show increased ability to colonize different hosts in the lab. Finally, we predict that the presence of environmentally modifying secretions will reduce the growth rate of specialist pathogens (relative to environmental modifier strains), giving strains that produce them an advantage over specialists in co-infections.

Both theory and bioinformatic analyses suggest that genes coding for social secretions are frequently associated with mobile genetic elements^14^,^30^. Combined with our results, this suggests that monitoring for the acquisition of large numbers of secreted proteins via horizontal gene transfer may help predict which pathogenic bacteria are likely to expand their host range to humans. Given that such monitoring of mobile genetic elements is frequently carried out to assess the spread of antibiotic resistance genes and virulence factors, such monitoring appears feasible to implement.

While our results highlight a previously unrecognized risk factor for host range expansion in pathogens, major challenges remain in integrating these results with previous work on risk factors for zoonosis. Previous large-scale studies on risk factors for zoonosis have largely focused on ecological and epidemiological factors (for example, wildlife diversity and human population density^5^) governing when and where zoonoses are likely to arise, rather than the organismal traits that govern which species are most likely to emerge as zoonoses^5^,^6^,^7^. Integrating these two perspectives on the risk factors for zoonosis will require targeted sampling of pathogen communities across a spectrum of ecological conditions to address how organismal traits and epidemiological factors combine to determine host range shifts.

Our ability to cooperatively modify and standardize our environment is commonly seen as a key element in humans’ success in colonizing virtually every terrestrial habitat on the earth^9^,^31^,^32^. Our results show that this mechanism for achieving generalism is not confined to humans and is widespread across bacteria. Sociality appears to be just as important for the spread of bacterial species to new niches as it has been in human history.

## Methods

### Comparative analysis

We gathered data on whether 191 species of bacteria that are pathogenic to humans are zoonotic (that is, can naturally transmit between humans and other vertebrate hosts, *n*=121) or azoonotic (that is, only infect humans, *n*=70) from a previous collation^4^. For these species we also collated their secretome (that is, proteins with an extracellular localization) and genome sizes from PSORTdb^24^. We included all available fully sequenced genomes within a species and their associated plasmids and took the mean value per strain within each species (Supplementary Table 1 contains all data used, and a list of genomes used is given in Supplementary Table 2). We used the SUPERFAMILY whole-genome-based phylogeny^25^, which has the advantage of minimizing the effects of horizontal gene transfer on the tree topology. For each species in our analysis, we used the type strain to produce the phylogeny.

We used a BPMM approach to test the effects of secretome and genome sizes on the probability that a species is zoonotic. Analyses were implemented in R using the package MCMCglmm^23^. We fit a model with a binomial error structure and genome and secretome size as predictors, and the phylogenetic covariance matrix as a random effect. We used a weakly informative Gelman prior for fixed effects^33^,^34^. We specified a prior of an inverse Wishart distribution for the random effect. The residual variance (overdispersion) was fixed to 1, as this cannot be estimated with binary data. Parameter estimates were subsequently scaled under the assumption that the true residual variance is 0. We ran the analysis for 3,000,000 iterations with a burn-in of 500,000 and thinning interval of 1,000 to minimize autocorrelation in the chains. We used the Gelman–Rubin test^35^,^36^, as well as visual inspection of traces, on three independent chains to ensure model convergence. Statistics quoted are modes and 95% CIs for the posterior distributions. Code for prior and model specification was as follows: Prior <- list(B=list(mu=c(0,0,0), V=gelman.prior(~secretome_size+genome_size, data=mydata, scale=1+1+pi^2/3)), R=list(V=1, fix=1), G=list(G1=list(V=diag(1)*0.1, nu=1))), Model <- MCMCglmm(zoonotic~secretome_size+genome_size, family=“categorical”, data=mydata, prior=Prior, pedigree=tree, scale=F, nitt=3000000, burnin=500000, thin=1000, verbose=F, slice=T, nodes=“TIPS”).

### Theoretical model

We use the framework of a two-host species epidemiological model to examine the scenarios in which a environmental modifier strategy is favoured. We first describe our model for the intra-host dynamics of each strain, before turning our attention to the epidemiological dynamics.

We consider four possible strategies that a pathogen can take; they can be a specialist on host species *H*_1_ or *H*_2_ (*S*_1_ and *S*_2_), a classical generalist (*G*) or a environmental modifier (*EM*). A classical generalist has growth *g* in host species *H*_1_ and *H*_2_. A specialist has growth rate *s* in the host species they specialize on. A specialist’s growth rate is 0 in the alternative host species. We assume that *s*>*g*, that is, there is a cost of generalism. The environmental modifier strategy attempts to modify the environment they experience in species *H*_1_ and *H*_2_ to a common type of environment to which they are adapted. It has a frequency-dependent growth rate of *bEM*−*c* (where *EM* is the frequency of the environmental modifier strain within the focal host) when it infects either host species *H*_1_ or *H*_2_. The environmental modifier modifies the current host environment towards a modified environment to extent *EM*, thus receiving a growth benefit of *bEM*, with a cost of *c* for investment in environmental modification. When in competition with a environmental modifier within a host a generalist will now have growth rate *g*(1−*EM*), as the environmental modifier modifies the host to species *H*_*X*_ as it increases in frequency. Similarly, a specialist co-infecting its preferred host species with a environmental modifier will have growth rate *s*(1−*EM*).

### Within-host dynamics

We use the replicator equation^26^ to model the within-host dynamics of these strains. The replicator equation can be written as


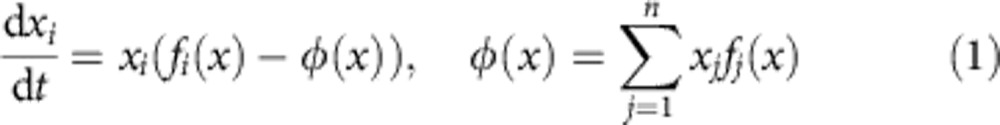


where *x*_*i*_ is the proportion of individuals within the focal host belonging to strain *i*, *x* is a vector of the frequencies of each strain within the focal host, *f*_*i*_(*x*) is the growth rate of strain *i* given strain frequencies *x* and *ϕ*(*x*) is the mean growth rate of the strains within the focal host.

Using our assumptions and equation (1) we can now write the dynamics of our three strategies in host species *H*_*i*_ as


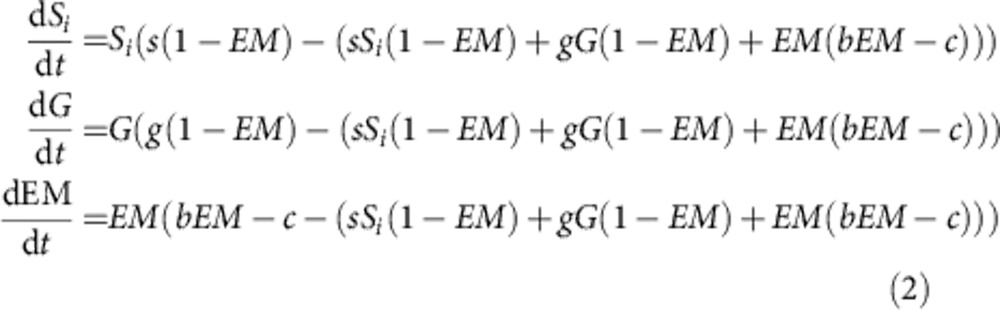


where *EM*, *G*, *S*_1_ and *S*_2_ are the frequencies of each strain within the focal host. As we will make an assumption of superinfection in our epidemiological model (see below), we need to only consider pairwise competition between strain types within a host. Also, as specialists have a growth rate of 0 in the alternative host, we need not consider this scenario.

Let us first consider competition between a specialist in its preferred host species and generalist. Setting *EM*=0 and *S*_*i*_+*G*=1 a standard stability analysis^37^ shows that *S*_i_*=1 is the only stable equilibrium as long as *s*>*g*, meaning that a specialist in its preferred host will always outcompete a classical generalist. When a specialist competes with a environmental modifier within a host (that is, setting *G*=0 and *S*_*i*_+*EM*=1) there are two possible stable equilibria at EM*=0 and EM*=1, separated by a repeller at *EM*=(*c*+*s*)/(*b*+*s*). If *EM*>(*c*+*s*)/(*b*+*s*) then environmental modifiers sweep to fixation and the equilibrium is EM*=1, while if *EM*<(*c*+*s*)/(*b*+*s*), specialists win out and the equilibrium is EM*=0. Similarly, when generalists and environmental modifiers compete within a host (*S*_*i*_=0, *G*+*EM*=1) there are two stable equilibria at EM*=0 and EM*=1, separated by a repeller at *EM*=(*c*+*g*)/(*b*+*g*). We note that these dynamics are similar to those of the classic stag-hunt game^38^, and a previous analysis of immune system provocation by pathogens to exclude competitors^12^. We can then generate the following rules from these within-host dynamics for inclusion in our epidemiological model:

1. *S*_*i*_ never infects host species *H*_*j*_, where *j*≠*i*.
2. *S*_*i*_ always outcompetes *G* in host species *H*_*i*_.
3. *EM* outcompetes *S*_*i*_ in host species *H*_*i*_ with probability 1−(*c*+*s*)/(*b*+*s*), while *S*_*i*_ outcompetes *EM* with probability (*c*+*s*)/(*b*+*s*).
4. *EM* outcompetes *G* in either host species with probability 1−(*c*+*g*)/(*b*+*g*), while *G* outcompetes *EM* with probability (*c*+*g*)/(*b*+*g*).

### Epidemiological dynamics

We use a susceptible–infected–susceptible model for the epidemiological dynamics. We stress that this is the simplest possible description of the epidemiological dynamics and will not hold for most bacterial species, many of which will show environmental growth. However, this model allows us to gain some insights into the epidemiological consequences of environmental modification, while remaining tractable. We also stress that the results of our model for within-host competition hold regardless of these epidemiological assumptions.

We will assume that within-host dynamics occur on much faster timescale than the epidemiological dynamics so that strain replacement occurs instantaneously on the epidemiological timescale and co-infection can be ignored (that is, a superinfection model). We can write the generic dynamics for a single strain in susceptible–infected–susceptible model with two host species, under the assumption that both host species show identical epidemiological properties, as


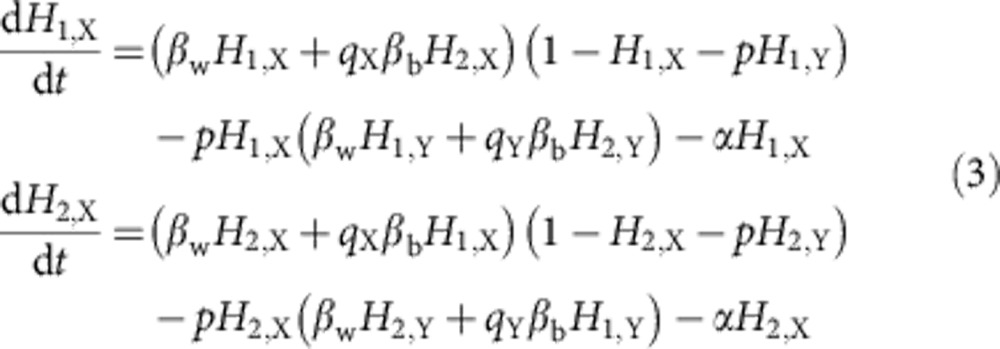


where, *H*_*i*,*Z*_ is the proportion of host species *i* infected with strain *Z*, *α* is the clearance rate of infections, *β*_w_ is the contact rate within a host species, *β*_b_ is the contact rate between the host species, *p* is the probability that strain *Y* outcompetes strain *X* within a host and *q*_*Z*_∈{0,1} denotes whether strain *Z* can infect both host species. In each differential equation the first term captures the spread of strain *X* to new hosts of species *i*, the second term captures replacement of strain *X* in host species *i* by strain *Y* and the third term captures the clearance of infections.

Combining this model framework with the assumptions and results of our within-host competition model we can write the epidemiological dynamics as 


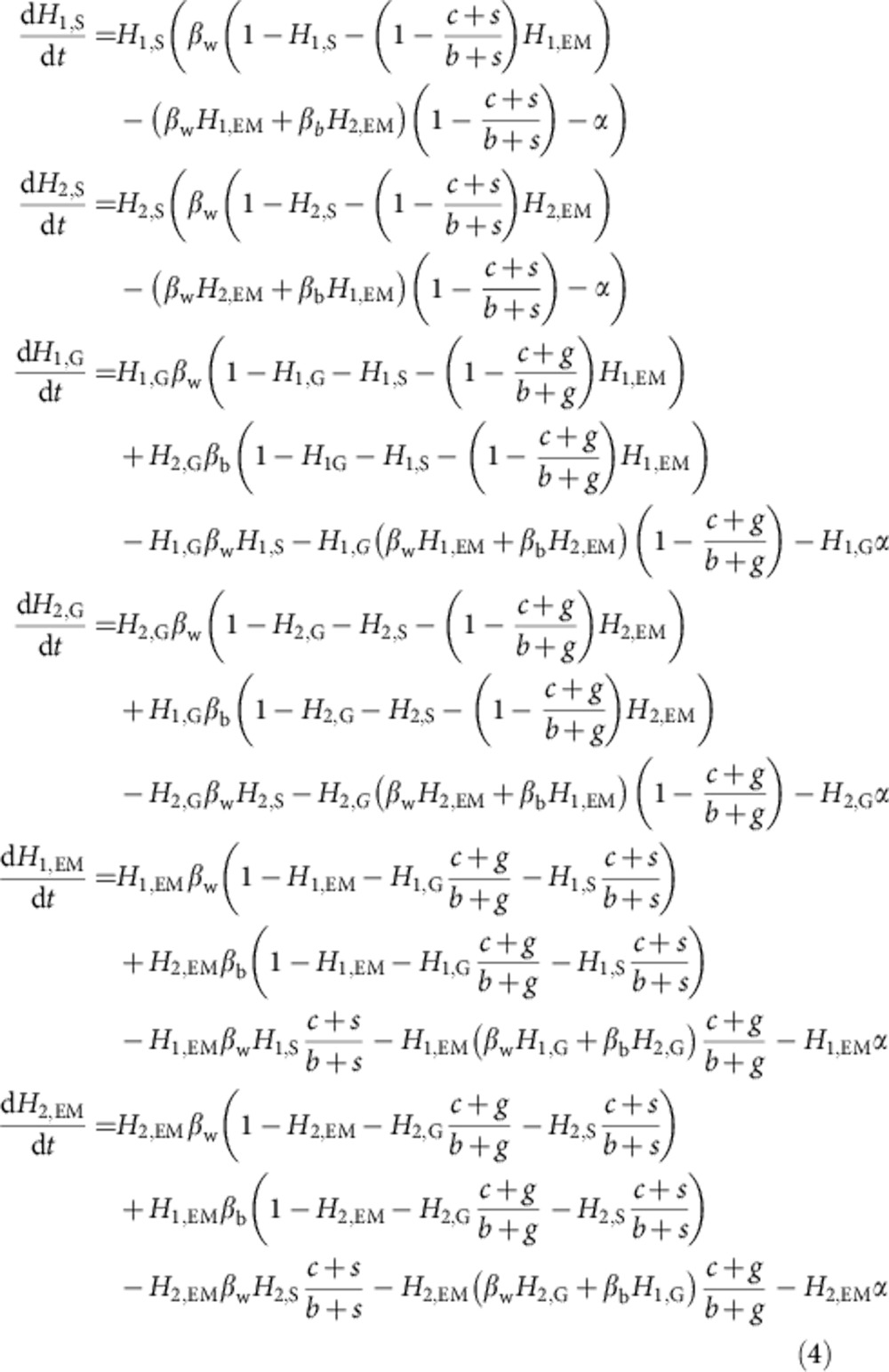


where, *H*_*i*,*Z*_ is again the proportion of host species *i* infected with strain type *Z*, and all other parameters are as above.

### Invading a population of specialists

We first consider the potential for both classical generalists and environmental modifiers to invade a population of specialists. First setting *H*_1,G_=*H*_2,G_=*H*_1,EM_=*H*_2,EM_=0, the stable frequencies of specialists are 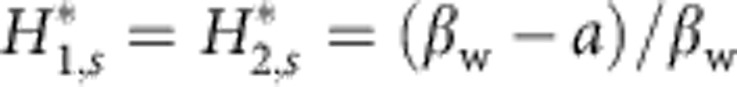, assuming that *β*_w_>*α* (that is, that specialists can exist). We now consider the invasion of rare classical generalists and environmental modifiers into this population of specialists. The key epidemiological condition for the invasion of a strain is that their ‘reproductive number’, *R*_0_>1 (ref. 39). We consider the scenario where the strain of interest is introduced from rarity in one of the host species (which host species is irrelevant as we assume they have identical epidemiological properties). We can write the conditions for invasion of classical generalists and environmental modifiers as


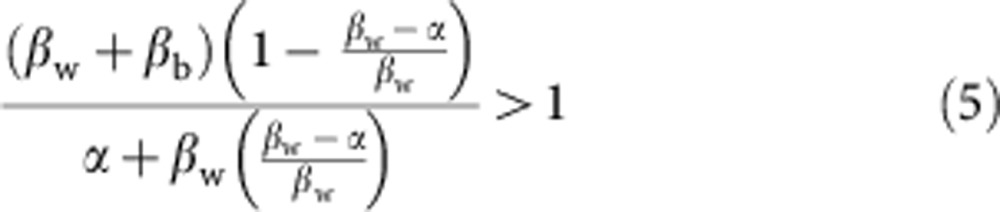


and


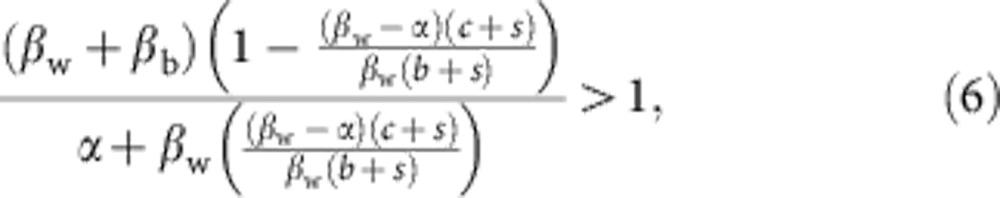


respectively. Here the numerators are the rate of spread of each strain. In the case of the classical generalists, this is simply the sum of the within- and between-host species contact rate times the proportion of hosts not currently infected with specialists. However, in the case of environmental modifiers, the number of specialist hosts is weighted by (*c*+*s*)/(*b*+*s*) as environmental modifiers can outcompete a specialist within a host with probability 1−(*c*+*s*)/(*b*+*s*). The denominators represent the loss of infections by the invading strain owing to clearance of infections and superinfection by specialists. In the case of classical generalists, this is simply the clearance rate of infection plus the within-host species transmission rate times the proportion of hosts infected by specialists. Again, however, in the case of environmental modifiers the number of specialist hosts is weighted by (*c*+*s*)/(*b*+*s*) as environmental modifiers can outcompete a specialist within a host with probability 1−(*c*+*s*)/(*b*+*s*). These two conditions (equations 5 and 6) are equivalent whenever *b*=*c*, while the condition for environmental modifiers to invade is more easily satisfied than that for classical generalists whenever *b*>*c*, that is, whenever environmental modification has a net positive effect on growth rate in a single strain infection.

### Invading a population of generalists

We now consider the conditions for invasion of a environmental modifier strain into a population of classical generalists. First setting *H*_1,S_=*H*_2,S_=*H*_1,EM_=*H*_2,EM_=0, the stable frequency of classical generalists is 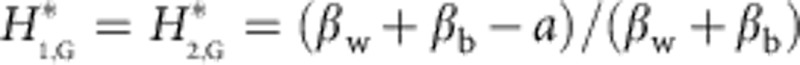, assuming that *β*_w_+*β*_b_>*α* (that is, that classical generalists can exist). We can now calculate the *R*_0_ for a environmental modifier strain invading the population of classical generalists as


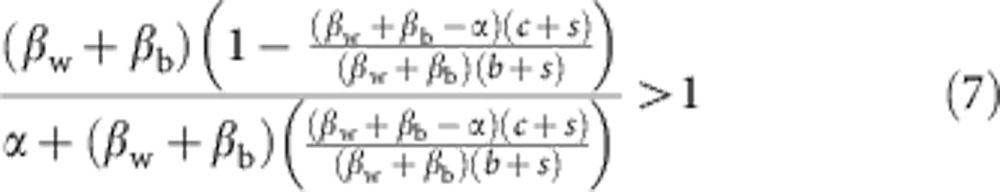


which simplifies to


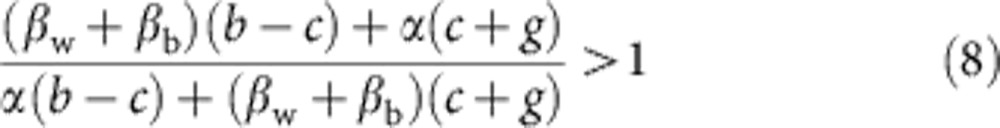


and gives the condition


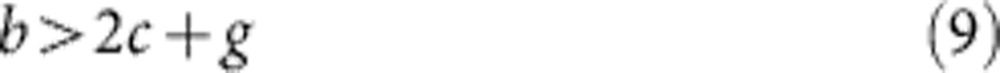


for the invasion of environmental modifiers into a population of classical generalists, meaning that for sufficiently high benefits, environmental modifiers can invade a population of classical generalists from rarity.

### A note on the problem of cheaters

The strategy of cooperative environmental modification that we have examined is in principle susceptible to cheaters that are adapted to the modified environment that environmental modifiers create but do not invest in, and hence do not pay a cost for its production. This problem of how cooperation can survive in the face of such cheating has received considerable theoretical and empirical attention and a number of solutions exist^13^. Regulatory control of these traits may be designed such that they are only expressed when costs are limited and/or benefits are maximized^40^,^41^. Population structure, either within a host or among hosts, will also favour cooperation by ensuring cooperative strains encounter each other more frequently^13^. In addition, there may be frequency dependence between cheaters and cooperators, leading to a mix of both strains at equilibrium^42^.

Although the potential of cheaters to undermine the evolution of cooperative traits involved in environmental modification is an evolutionary problem of great interest, it does not pose a major obstacle for our analyses. First, the same factors that favour the environmental modification strategy in our model (high benefits and low costs) also limit the evolutionary potential for cheating^13^. Second, in our comparative analysis we considered the number of genes coding for secretions that a bacteria possesses. Given that these genes exist, it is unlikely that cheaters have purged the population of all cooperation. Although cheaters may prove a significant obstacle in the evolution of cooperative environmental modification, this does not denigrate our result that those bacteria that successfully evolve environmental modification can achieve host generalism.

## Author contributions

L.M. and S.P.B. developed the theoretical model. L.M. and M.V. collated and analysed the data. L.M. drafted the manuscript. All authors contributed to conceptual development, study design and manuscript revision.

## Additional information

**How to cite this article:** McNally, L. *et al.* Cooperative secretions facilitate host range expansion in bacteria. *Nat. Commun.* 5:4594 doi: 10.1038/ncomms5594 (2014).

## Supplementary Material

Supplementary Information Supplementary Figure 1 and Supplementary Tables 1-2

## Figures and Tables

**Figure 1:**
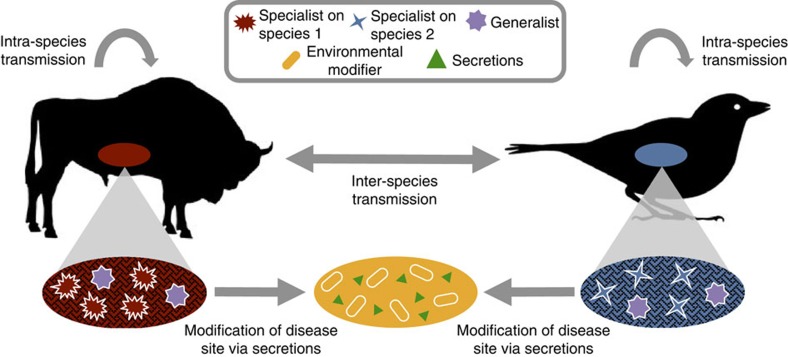
Environment-modifying secretions as a route to host generalism. We consider a scenario where pathogens can potentially transmit both within and among host species. Whereas specialists match their hosts closely (matching colours), generalists that infect multiple hosts are expected to have intermediate phenotypes (intermediate colour), meaning that they will lose to specialists during co-infections. While environmental modifiers may lose to specialists and generalists in the unmodified disease site, they can potentially invade by modifying this environment (transitions from red/blue to yellow) via the production of costly secretions (green triangles) that simplify the environment (loss of patterns). Specialists and classical generalists are not adapted to this modified environment, leading to their exclusion. While specialists and classical generalists are expected to show complex adaptations to their host(s) (complex shapes), environmental modifiers are expected to show simpler adaptations (simple shape), instead relying on secretions that modify and simplify their environment.
