# Supplementary material for: Cooperative secretions facilitate host range expansion in bacteria
Source: Nat Commun. 2014 Aug 5;5:4594. doi: 10.1038/ncomms5594 (PMC4143932; doi:10.1038/ncomms5594)
Supplement: Supplementary Information — Supplementary Figure 1 and Supplementary Tables 1-2 [file ncomms5594-s1.pdf]

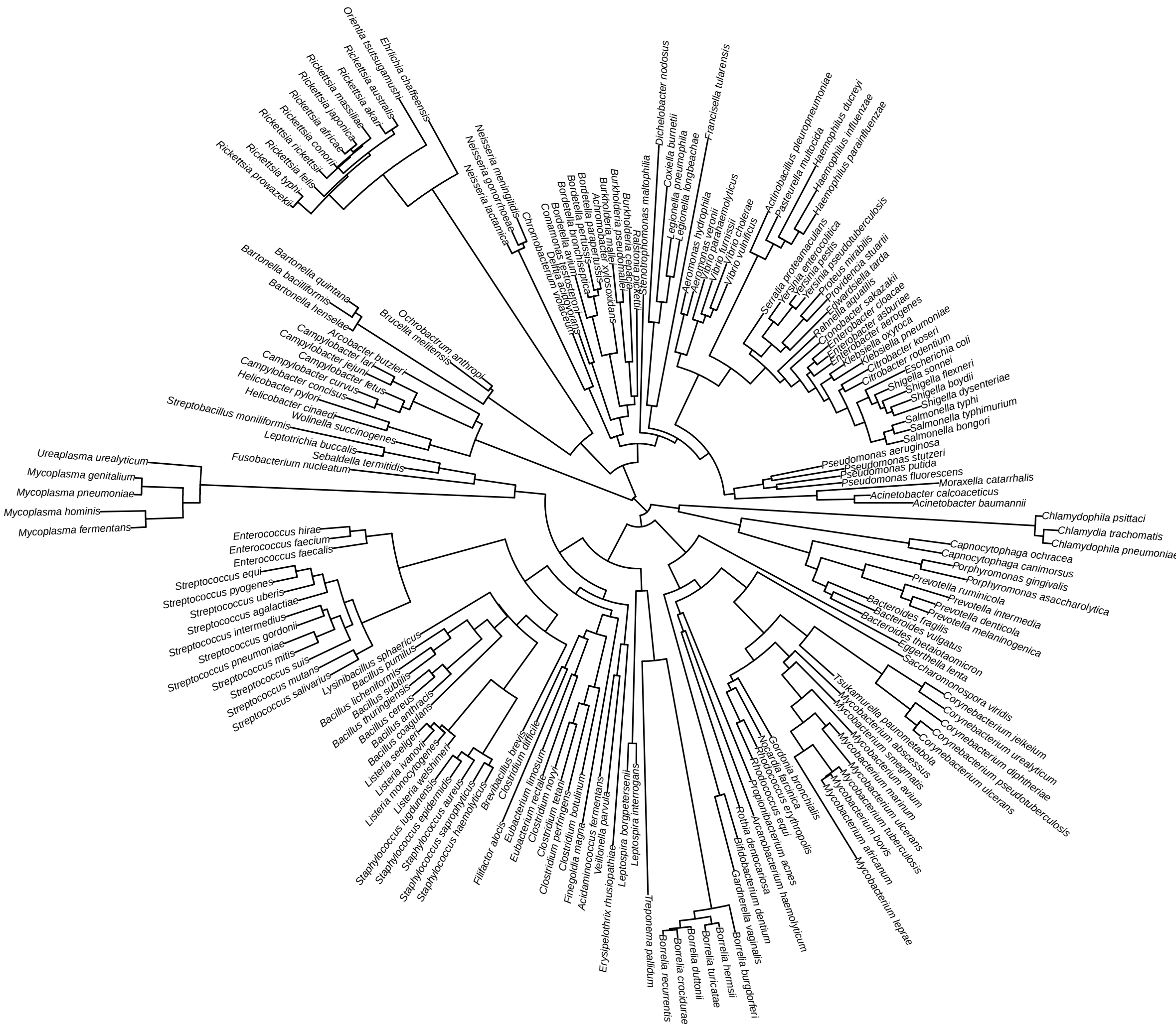

Supplementary figure 1: The untransformed phylogeny used in our Bayesian phylogenetic mixed model.

Supplementary table 1. Data used in comparative analysis

Species	Zoonotic	Genome size	Secretome size
Achromobacter xylosoxidans	0	6601.5	53.5
Acidaminococcus fermentans	0	2026.0	10.0
Acinetobacter baumannii	0	3688.7	38.8
Acinetobacter calcoaceticus	0	3599.0	42.0
Actinobacillus pleuropneumoniae	1	2063.3	12.0
Aeromonas hydrophila	1	4277.5	52.5
Aeromonas veronii	0	4028.0	44.0
Arcanobacterium haemolyticum	1	1731.0	16.0
Arcobacter butzleri	1	2205.3	15.0
Bacillus anthracis	1	5576.7	120.4
Bacillus cereus	1	5475.5	117.3
Bacillus coagulans	1	3130.5	46.5
Bacillus licheniformis	1	4196.7	75.3
Bacillus pumilus	0	3678.0	62.0
Bacillus subtilis	1	4098.5	86.9
Bacillus thuringiensis	0	6054.7	140.7
Bacteroides fragilis	1	4382.0	37.3
Bacteroides thetaiotaomicron	0	4816.0	55.0
Bacteroides vulgatus	0	4065.0	35.0
Bartonella bacilliformis	0	1283.0	8.0
Bartonella henselae	1	1488.0	10.0
Bartonella quintana	0	1172.5	3.5
Bifidobacterium dentium	0	2129.0	20.0
Bordetella avium	0	3381.0	35.0
Bordetella bronchiseptica	1	4799.0	35.7
Bordetella parapertussis	1	4184.5	29.0
Bordetella pertussis	0	3423.0	20.7
Borrelia burgdorferi	1	1271.2	7.0
Borrelia crocidurae	1	1470.0	7.0
Borrelia duttonii	0	1305.0	11.0
Borrelia hermsii	1	877.0	7.0
Borrelia recurrentis	0	990.0	7.0
Borrelia turicatae	1	818.0	7.0
Brevibacillus brevis	0	5947.0	97.0
Brucella melitensis	1	3219.7	18.8
Burkholderia cepacia	0	4614.5	54.5
Burkholderia mallei	1	5284.0	68.8
Burkholderia pseudomallei	1	6528.3	107.4
Campylobacter concisus	0	1985.0	25.0
Campylobacter curvus	0	1931.0	23.0
Campylobacter fetus	1	1719.0	23.0
Campylobacter jejuni	1	1685.7	18.8
Campylobacter lari	1	1545.0	14.0
Capnocytophaga canimorsus	1	2404.0	12.0

Capnocytophaga ochracea	0	2171.0	7.0
Chlamydia trachomatis	1	909.3	15.8
Chlamydophila pneumoniae	1	1090.2	18.6
Chlamydophila psittaci	1	982.0	16.6
Chromobacterium violaceum	1	4407.0	92.0
Citrobacter koseri	1	5008.0	72.0
Citrobacter rodentium	1	4913.0	67.0
Clostridium botulinum	1	3346.4	78.1
Clostridium difficile	1	2929.4	29.0
Clostridium novyi	1	2315.0	34.0
Clostridium perfringens	1	2721.7	52.3
Clostridium tetani	1	2432.0	31.0
Comamonas testosteroni	0	4799.0	46.0
Corynebacterium diphtheriae	0	2276.0	27.1
Corynebacterium jeikeium	0	2120.0	27.0
Corynebacterium pseudotuberculosis	1	2078.1	24.8
Corynebacterium ulcerans	1	2287.7	22.7
Corynebacterium urealyticum	0	1978.5	24.5
Coxiella burnetii	1	1935.0	7.4
Cronobacter sakazakii	1	4049.0	53.0
Delftia acidovorans	0	6040.0	73.0
Dichelobacter nodosus	1	1280.0	10.0
Edwardsiella tarda	1	3416.3	30.3
Eggerthella lenta	1	3070.0	17.0
Ehrlichia chaffeensis	1	1105.0	10.0
Enterobacter aerogenes	1	5205.5	61.0
Enterobacter asburiae	1	4612.0	62.0
Enterobacter cloacae	1	4522.8	62.2
Enterococcus faecalis	1	2913.6	39.6
Enterococcus faecium	1	2976.5	32.5
Enterococcus hirae	1	2755.0	50.0
Erysipelothrix rhusiopathiae	1	1736.0	11.5
Escherichia coli	1	4751.3	62.2
Eubacterium limosum	1	4579.0	69.0
Eubacterium rectale	1	3239.7	61.0
Filifactor alocis	0	1641.0	16.0
Finegoldia magna	0	1813.0	20.0
Francisella tularensis	1	1626.2	19.1
Fusobacterium nucleatum	1	2107.0	2.5
Gardnerella vaginalis	0	1313.3	15.7
Gordonia bronchialis	0	4696.0	54.0
Haemophilus ducreyi	0	1717.0	9.0
Haemophilus influenzae	0	1735.5	13.8
Haemophilus parainfluenzae	0	1975.0	17.0
Helicobacter cinaedi	0	2122.0	21.5
Helicobacter pylori	1	1604.6	25.7

Klebsiella oxytoca	1	5755.5	60.5
Klebsiella pneumoniae	1	4723.8	30.3
Legionella longbeachae	0	3470.0	47.0
Legionella pneumophila	0	2815.5	39.9
Leptospira borgpetersenii	1	2912.5	24.5
Leptospira interrogans	1	3690.3	62.7
Leptotrichia buccalis	1	2220.0	8.0
Listeria ivanovii	1	2650.0	27.0
Listeria monocytogenes	1	2922.4	34.4
Listeria seeligeri	1	2710.0	31.0
Listeria welshimeri	1	2774.0	29.0
Lysinibacillus sphaericus	0	4771.0	73.0
Moraxella catarrhalis	0	1886.0	8.0
Mycobacterium abscessus	1	5063.0	66.0
Mycobacterium africanum	1	3830.0	62.0
Mycobacterium avium	1	4598.7	49.7
Mycobacterium bovis	1	3980.4	59.0
Mycobacterium leprae	1	1604.5	17.0
Mycobacterium marinum	1	5452.0	86.0
Mycobacterium smegmatis	1	6781.7	74.3
Mycobacterium tuberculosis	1	3945.1	65.2
Mycobacterium ulcerans	1	4241.0	49.0
Mycoplasma fermentans	1	913.3	14.3
Mycoplasma genitalium	0	494.6	5.4
Mycoplasma hominis	0	523.0	4.0
Mycoplasma pneumoniae	0	659.3	7.3
Neisseria gonorrhoeae	0	2290.7	8.7
Neisseria lactamica	0	1972.0	10.0
Neisseria meningitidis	0	1953.1	11.6
Nocardia farcinica	1	5936.0	64.0
Ochrobactrum anthropi	0	4799.0	31.0
Orientia tsutsugamushi	1	1574.5	10.0
Pasteurella multocida	1	2150.3	11.8
Porphyromonas asaccharolytica	1	1699.0	22.0
Porphyromonas gingivalis	1	2072.0	5.7
Prevotella denticola	0	2386.0	19.0
Prevotella intermedia	0	2266.0	20.0
Prevotella melaninogenica	1	2296.0	23.0
Prevotella ruminicola	1	2763.0	45.0
Propionibacterium acnes	1	2294.9	31.5
Proteus mirabilis	1	3558.5	68.0
Providencia stuartii	1	4099.0	95.0
Pseudomonas aeruginosa	1	5899.6	67.3
Pseudomonas fluorescens	0	5890.2	66.6
Pseudomonas putida	0	5506.4	40.5
Pseudomonas stutzeri	0	4170.8	35.3

Rahnella aquatilis	0	1658.0	24.2
Ralstonia pickettii	0	5156.5	68.5
Rhodococcus equi	1	4512.0	55.0
Rhodococcus erythropolis	0	6130.5	76.5
Rickettsia africae	1	1041.0	9.0
Rickettsia akari	1	1258.0	10.0
Rickettsia australis	1	1261.0	7.0
Rickettsia conorii	1	1374.0	12.0
Rickettsia felis	1	1512.0	16.0
Rickettsia japonica	1	971.0	7.0
Rickettsia massiliae	1	1093.5	8.5
Rickettsia prowazekii	1	867.7	4.8
Rickettsia rickettsii	1	1343.5	7.0
Rickettsia typhi	1	838.3	4.0
Rothia dentocariosa	0	2217.0	30.0
Saccharomonospora viridis	0	3828.0	42.0
Salmonella bongori	1	4316.5	64.0
Salmonella typhi	0	4621.5	67.8
Salmonella typhimurium	1	4525.0	71.0
Sebaldella termitidis	0	4150.0	15.0
Serratia proteamaculans	0	4942.0	62.0
Shigella boydii	1	4419.0	67.5
Shigella dysenteriae	1	4501.0	49.0
Shigella flexneri	1	4329.8	61.3
Shigella sonnei	1	4941.0	69.5
Staphylococcus aureus	1	2526.7	84.6
Staphylococcus epidermidis	1	2505.0	61.0
Staphylococcus haemolyticus	1	2692.0	64.0
Staphylococcus lugdunensis	0	2457.5	38.5
Staphylococcus saprophyticus	0	2514.0	41.0
Stenotrophomonas maltophilia	0	4175.3	51.7
Streptobacillus moniliformis	1	1442.0	2.0
Streptococcus agalactiae	1	1972.6	24.6
Streptococcus equi	1	1962.5	27.5
Streptococcus gordonii	0	2051.0	26.0
Streptococcus intermedius	1	1765.0	20.7
Streptococcus mitis	0	2004.0	30.0
Streptococcus mutans	0	1913.5	25.8
Streptococcus pneumoniae	1	2034.7	41.7
Streptococcus pyogenes	1	1808.3	27.6
Streptococcus salivarius	0	1982.3	41.0
Streptococcus suis	1	1998.8	15.9
Streptococcus uberis	1	1762.0	15.0
Treponema pallidum	0	1026.0	9.4
Tsukamurella paurometabola	0	4242.0	44.0
Ureaplasma urealyticum	1	646.0	9.0

Veillonella parvula	0	1844.0	8.0
Vibrio cholerae	0	3721.3	45.6
Vibrio furnissii	1	4455.0	60.0
Vibrio parahaemolyticus	1	4647.3	65.5
Vibrio vulnificus	1	4673.0	53.0
Wolinella succinogenes	0	2042.0	18.0
Yersinia enterocolitica	1	4176.7	76.3
Yersinia pestis	1	4071.8	76.3
Yersinia pseudotuberculosis	1	4197.8	75.3

Supplementary table 2. Accession numbers of genomes used in this study

Achromobacter xylosoxidans
NC_014640.1
NC_014641.1
NC_014642.1
NC_021285.1

Acidaminococcus fermentans
NC_013740.1

Acinetobacter baumannii
NC_017162.1
NC_017163.1
NC_017164.1
NC_011585.1
NC_011586.1
NC_011595.1
NC_010605.1
NC_010606.1
NC_010611.1
NC_009083.1
NC_009084.1
NC_009085.1
NC_010401.1
NC_010402.1
NC_010404.1
NC_010403.1
NC_010410.1
NC_021727.1
NC_021728.1
NC_021726.1
NC_021734.1
NC_021733.1
NC_021730.1
NC_021731.1
NC_021732.1
NC_021729.1
NC_020525.1
NC_020547.1
NC_017847.1
NC_017848.1
NC_020524.1
NC_017171.1
NC_017172.1
NC_010395.1
NC_010396.1
NC_010398.1
NC_010400.1
NC_017387.1
NC_017165.1
NC_017166.1
NC_018706.1
NC_010410.1
NC_021727.1
NC_021728.1
NC_021726.1
NC_021734.1
NC_021733.1
NC_021730.1
NC_021731.1
NC_021732.1

Acinetobacter calcoaceticus
NC_016603.1

Actinobacillus pleuropneumoniae
NC_009053.1
NC_010278.1
NC_010941.1
NC_010942.1
NC_010940.1
NC_010939.1

Aeromonas hydrophila
NC_021290.1
NC_008570.1

Aeromonas veronii
NC_015424.1

Arcanobacterium haemolyticum
NC_014218.1

Arcobacter butzleri
NC_021878.1
NC_017187.1
NC_009850.1

Bacillus anthracis
NC_014335.1
NC_014333.1
NC_014331.1
NC_014332.1
NC_007322.2
NC_007323.3
NC_007530.2
NC_012656.1
NC_012655.1
NC_012659.1
NC_003980.1
NC_003981.1
NC_003997.3
NC_012579.1
NC_012577.1
NC_012581.1
NC_017729.1
NC_017726.1
NC_017727.1
NC_005945.1

Bacillus cereus
NC_012473.1
NC_012472.1
NC_011654.1
NC_011655.1
NC_011657.1
NC_011656.1
NC_011658.1
NC_011771.1
NC_011777.1
NC_011776.1
NC_011773.1
NC_005707.1
NC_003909.8
NC_004721.2
NC_004722.1
NC_011725.1
NC_007103.1
NC_007104.1
NC_007105.1
NC_007106.1
NC_007107.1
NC_006274.1
NC_016779.1
NC_016780.1
NC_016794.1
NC_018491.1
NC_018492.1
NC_018493.1
NC_018499.1

NC_018494.1
NC_011774.1
NC_011775.1
NC_011772.1
NC_016772.1
NC_016773.1
NC_016793.1
NC_016774.1
NC_016792.1
NC_016771.1
NC_011973.1
NC_011971.1
NC_011969.1
NC_009673.1
NC_009674.1

Bacillus coagulans
NC_015634.1
NC_016023.1

Bacillus licheniformis
NC_021362.1
NC_006270.3
NC_006322.1

Bacillus pumilus
NC_009848.1

Bacillus subtilis
NC_014976.1
NC_018520.1
NC_017194.1
NC_017196.1
NC_014479.1
NC_016047.1
NC_020507.1
NC_017195.1
NC_000964.3
NC_020832.1
NC_019896.1
NC_020244.1

Bacillus thuringiensis
NC_014171.1
NC_014172.1
NC_018877.1
NC_018880.1
NC_018885.1
NC_018878.1
NC_018884.1
NC_018883.1
NC_018879.1
NC_018882.1
NC_018881.1
NC_018886.1
NC_018500.1
NC_018486.1
NC_018501.1
NC_018487.1
NC_018488.1
NC_018502.1
NC_018489.1
NC_018503.1
NC_018490.1
NC_018516.1
NC_018509.1
NC_018510.1
NC_018517.1
NC_018511.1
NC_018512.1
NC_018508.1
NC_018693.1
NC_018686.1
NC_018687.1
NC_018688.1
NC_018689.1
NC_018694.1
NC_018684.1
NC_018685.1
NC_017208.1
NC_017202.1
NC_017209.1
NC_017203.1
NC_017204.1
NC_017210.1
NC_017205.1
NC_017211.1
NC_017206.1
NC_017207.1
NC_017212.1
NC_017200.1
NC_017201.1
NC_017199.1
NC_006578.1
NC_005957.1
NC_020240.1
NC_020250.1
NC_020243.1
NC_020249.1
NC_020239.1
NC_020241.1
NC_020242.1
NC_020238.1
NC_020377.1
NC_020378.1
NC_020379.1
NC_020381.1
NC_020382.1
NC_020383.1
NC_020384.1
NC_020385.1
NC_020390.1
NC_020391.1
NC_020392.1
NC_020393.1
NC_020394.1
NC_020380.1
NC_020376.1
NC_008598.1
NC_008600.1

Bacteroides fragilis
NC_016776.1
NC_006873.1
NC_003228.3
NC_006297.1
NC_006347.1

Bacteroides thetaiotaomicron
NC_004703.1
NC_004663.1

Bacteroides vulgatus
NC_009614.1

Bartonella bacilliformis
NC_008783.1

Bartonella henselae
NC_005956.1

Bartonella quintana
NC_018533.1
NC_005955.1

Bifidobacterium dentium
NC_013714.1

Bordetella avium

NC_010645.1

Bordetella bronchiseptica
NC_019382.1
NC_018829.1
NC_002927.3

Bordetella parapertussis
NC_002928.3
NC_018830.1
NC_018828.1

Bordetella pertussis
NC_018518.1
NC_017223.1
NC_002929.2

Borrelia burgdorferi
NC_000948.1
NC_000949.1
NC_000950.1
NC_000951.1
NC_000952.1
NC_000953.1
NC_000954.1
NC_001904.1
NC_001849.2
NC_000955.2
NC_001850.1
NC_001851.2
NC_001852.1
NC_001853.1
NC_001854.1
NC_001855.1
NC_001856.1
NC_000957.1
NC_001857.2
NC_000956.1
NC_001318.1
NC_022048.1
NC_017403.1
NC_017395.1
NC_017394.1
NC_017428.1
NC_017393.1
NC_017396.1
NC_017427.1
NC_017425.1
NC_017426.1
NC_017397.1
NC_017410.1
NC_017409.1
NC_017404.1
NC_017405.1
NC_017407.1
NC_017406.1
NC_017408.1
NC_017412.1
NC_017411.1
NC_017413.1
NC_013129.1
NC_017418.1
NC_017401.1
NC_017424.1
NC_017423.1
NC_017400.1
NC_017398.1
NC_017422.1
NC_017402.1
NC_017399.1
NC_017417.1
NC_017420.1
NC_017419.1
NC_017416.1
NC_017415.1
NC_017414.1
NC_017421.1
NC_013130.1
NC_001903.1
NC_011724.1
NC_011731.1
NC_011735.1
NC_011720.1
NC_011736.1
NC_011722.1
NC_011782.1
NC_011783.1
NC_011780.1
NC_011779.1
NC_011781.1
NC_011785.1
NC_011778.1
NC_011784.1
NC_011728.1

Borrelia crocidurae
NC_017808.1
NC_017774.1
NC_017775.1
NC_017776.1
NC_017777.1
NC_017778.1
NC_017779.1
NC_017780.1
NC_017781.1
NC_017782.1
NC_017783.1
NC_017784.1
NC_017785.1
NC_017786.1
NC_017787.1
NC_017788.1
NC_017789.1
NC_017794.1
NC_017795.1
NC_017796.1
NC_017797.1
NC_017798.1
NC_017799.1
NC_017800.1
NC_017801.1
NC_017802.1
NC_017809.1
NC_017810.1
NC_017811.1
NC_017812.1
NC_017813.1
NC_017814.1
NC_017815.1
NC_017816.1
NC_017817.1
NC_017818.1
NC_017819.1
NC_017820.1
NC_017821.1
NC_017822.1

Borrelia duttonii
NC_011224.1
NC_011226.1
NC_011247.1
NC_011257.1
NC_011259.1
NC_011261.1
NC_011265.1
NC_011245.1
NC_011262.1
NC_011264.1
NC_011248.1
NC_011249.1

NC_011250.1
NC_011251.1
NC_011254.1
NC_011256.1
NC_011229.1

Borrelia hermsii
NT_187136.1
NT_187138.1
NT_187137.1
NT_187139.1
NT_187140.1
NT_187141.1
NT_187142.1
NT_187143.1
NT_187144.1
NC_010673.1
NT_187145.1

Borrelia recurrentis
NC_011246.1
NC_011252.1
NC_011253.1
NC_011255.1
NC_011258.1
NC_011260.1
NC_011263.1
NC_011244.1

Borrelia turicatae
NC_008710.1

Brevibacillus brevis
NC_012491.1

Brucella melitensis
NC_003317.1
NC_003318.1
NC_012441.1
NC_012442.1
NC_007618.1
NC_007624.1
NC_017244.1
NC_017245.1
NC_017246.1
NC_017247.1
NC_017248.1
NC_017283.1

Burkholderia cepacia
NC_008391.1
NC_008392.1
NC_008385.1
NC_018513.1
NC_018514.1

Burkholderia mallei
NC_006348.1
NC_006349.2
NC_008836.1
NC_008835.1
NC_009080.1
NC_009079.1
NC_008785.1
NC_008784.1

Burkholderia pseudomallei
NC_017831.1
NC_017832.1
NC_009076.1
NC_009078.1
NC_007434.1
NC_007435.1
NC_009074.1
NC_009075.1
NC_018527.1
NC_018529.1
NC_006350.1
NC_006351.1
NC_021877.1
NC_021884.1
NC_012695.1

Campylobacter concisus
NC_009796.1
NC_009795.1
NC_009802.1

Campylobacter curvus
NC_009715.1

Campylobacter fetus
NC_008599.1

Campylobacter jejuni
NC_021834.1
NC_003912.7
NC_009707.1
NC_022362.1
NC_022352.1
NC_022351.1
NC_022353.1
NC_022354.1
NC_008790.1
NC_008770.1
NC_008787.1
NC_009839.1
NC_017279.1
NC_017284.1
NC_014802.1
NC_014801.1
NC_017280.1
NC_002163.1
NC_018521.1
NC_018709.2
NC_017281.1
NC_017282.1

Campylobacter lari
NC_012040.1
NC_012039.1

Capnocytophaga canimorsus
NC_015846.1

Capnocytophaga ochracea
NC_013162.1

Chlamydia trachomatis
NC_010287.1
NC_020966.1
NC_019272.1
NC_020939.1
NC_020946.1
NC_020944.1
NC_020979.1
NC_007430.1
NC_007429.1
NC_017437.1
NC_017438.1
NC_016798.1
NC_012686.1
NC_012687.1
NC_017434.1
NC_017435.1
NC_017436.1
NC_017433.1
NC_020967.1

NC_020986.1
NC_020943.1
NC_020958.1
NC_020968.1
NC_020959.1
NC_000117.1
NC_017431.1
NC_017439.1
NC_020971.1
NC_020947.1
NC_020969.1
NC_020987.1
NC_020942.1
NC_020960.1
NC_017952.1
NC_022107.1
NC_020964.1
NC_020988.1
NC_017951.1
NC_017953.1
NC_017440.1
NC_017430.1
NC_017432.1
NC_017429.1
NC_020941.1
NC_020961.1
NC_020970.1
NC_020962.1
NC_020940.1
NC_020989.1
NC_020511.1
NC_020551.1
NC_020512.1
NC_020513.1
NC_021892.1
NC_020965.1
NC_020963.1
NC_020929.1
NC_020951.1
NC_020972.1
NC_020982.1
NC_020973.1
NC_020952.1
NC_020937.1
NC_020930.1
NC_021049.1
NC_021052.1
NC_021051.1
NC_021050.1
NC_020938.1

NC_020983.1
NC_020945.1
NC_020984.1
NC_020933.1
NC_020980.1
NC_020934.1
NC_020948.1
NC_020977.1
NC_020949.1
NC_020978.1
NC_020981.1
NC_020936.1
NC_020950.1
NC_020935.1
NC_020953.1
NC_020932.1
NC_020954.1
NC_020976.1
NC_020955.1
NC_020975.1
NC_020985.1
NC_010280.2
NC_020931.1
NC_020956.1
NC_015744.1
NC_020974.1
NC_020957.1
NC_012627.1
NC_012631.1
NC_012625.1
NC_012626.1
NC_021890.1
NC_021888.1
NC_021895.1
NC_021891.1
NC_021896.1
NC_021889.1
NC_021898.1
NC_021899.1
NC_021897.1
NC_021887.1
NC_021893.1
NC_022119.1
NC_022109.1
NC_022108.1
NC_022117.1
NC_022106.1
NC_022118.1
NC_022120.1
NC_022110.1

NC_022121.1
NC_017441.1

Chlamydophila pneumoniae
NC_002179.2
NC_000922.1
NC_002491.1
NC_017285.1
NC_017286.1
NC_005043.1

Chlamydophila psittaci
NC_017289.1
NC_017292.1
NC_017290.1
NC_015470.1
NC_017287.1
NC_015217.1
NC_017288.1
NC_017291.1
NC_020248.1
NC_014797.1
NC_014796.1

Chromobacterium violaceum
NC_005085.1

Citrobacter koseri
NC_009794.1
NC_009793.1
NC_009792.1

Citrobacter rodentium
NC_013717.1
NC_013718.1
NC_013719.1
NC_013716.1

Clostridium botulinum
NC_009697.1
NC_009496.1
NC_009495.1
NC_009698.1
NC_012563.1
NC_010418.1
NC_010520.1
NC_010680.1
NC_018653.1

NC_018648.1
NC_010674.1
NC_010379.1
NC_010516.1
NC_012654.1
NC_012657.1
NC_012658.1
NC_015425.1
NC_015417.1
NC_015426.1
NC_015418.1
NC_015427.1
NC_015419.1
NC_010723.1
NC_017297.1
NC_017298.1
NC_009700.1
NC_009699.1
NC_017299.1

Clostridium difficile
NC_008226.1
NC_009089.1
NC_017176.1
NC_017177.1
NC_017179.1
NC_013315.1
NC_013316.1

Clostridium novyi
NC_008593.1

Clostridium perfringens
NC_008261.1
NC_008263.1
NC_008264.1
NC_008262.1
NC_003042.1
NC_003366.1

Clostridium tetani
NC_004565.1
NC_004557.1

Comamonas testosteroni
NC_013446.1

Corynebacterium diphtheriae
NC_016782.1
NC_016799.1
NC_016800.1
NC_016801.1
NC_016785.1
NC_016786.1
NC_016802.1
NC_016787.1
NC_016788.1
NC_016783.1
NC_002935.2
NC_016789.1
NC_016790.1

Corynebacterium jeikeium
NC_003080.1
NC_007164.1

Corynebacterium pseudotuberculosis
NC_017308.1
NC_017300.1
NC_017945.1
NC_017462.1
NC_016781.1
NC_017730.1
NC_016932.1
NC_017306.1
NC_017301.1
NC_017307.1
NC_018019.1
NC_014329.1
NC_017303.1
NC_017031.1
NC_017305.1

Corynebacterium ulcerans
NC_018101.1
NC_017317.1
NC_015683.1

Corynebacterium urealyticum
NC_010545.1
NC_020230.1

Coxiella burnetii
NC_011527.1
NC_011526.1
NC_011528.1
NC_009726.1
NC_009727.1
NC_010115.1
NC_010117.1
NC_004704.1
NC_002971.3

Cronobacter sakazakii
NC_017933.1
NC_020263.1
NC_020261.1
NC_020262.1
NC_020260.1

Delftia acidovorans
NC_010002.1

Dichelobacter nodosus
NC_009446.1

Edwardsiella tarda
NC_020796.1
NC_013509.1
NC_013508.1
NC_017309.1
NC_017318.1

Eggerthella lenta
NC_013204.1

Ehrlichia chaffeensis
NC_007799.1

Enterobacter aerogenes
NC_020180.1
NC_020182.1
NC_020181.1
NC_015663.1

Enterobacter asburiae
NC_015968.1
NC_015963.1
NC_015969.1

Enterobacter cloacae
NC_016514.1
NC_016515.1
NC_014618.1
NC_014121.1
NC_014107.1

NC_014108.1
NC_018405.1
NC_021046.1
NC_018079.1

Enterococcus faecalis
NC_017312.1
NC_017314.1
NC_017313.1
NC_017315.1
NC_018221.1
NC_018222.1
NC_018223.1
NC_017316.1
NC_019770.1
NC_004669.1
NC_004671.1
NC_004670.1
NC_004668.1

Enterococcus faecium
NC_017022.1
NC_017032.1
NC_017023.1
NC_017024.1
NC_021987.1
NC_021995.1
NC_021988.1
NC_021989.1
NC_021996.1
NC_021990.1
NC_021994.1
NC_017960.1
NC_017961.1
NC_017962.1
NC_017963.1
NC_020208.1
NC_020207.1

Enterococcus hirae
NC_018081.1
NC_015845.1

Erysipelothrix rhusiopathiae
NC_015601.1
NC_021354.1

Escherichia coli
NC_017627.1
NC_017626.1
NC_008253.1
NC_011748.1
NC_017631.1
NC_017629.1
NC_009837.1
NC_009838.1
NC_008563.1
NC_020163.1
NC_010468.1
NC_012967.1
NC_012971.2
NC_012947.1
NC_012892.2
NC_012759.1
NC_004431.1
NC_017625.1
NC_017638.1
NC_009787.1
NC_009791.1
NC_009789.1
NC_009788.1
NC_009790.1
NC_009786.1
NC_009801.1
NC_011745.1
NC_017721.1
NC_017723.1
NC_017722.1
NC_017724.1
NC_017633.1
NC_009800.1
NC_011741.1
NC_011750.1
NC_017628.1
NC_016902.1
NC_017660.1
NC_016904.1
NC_016903.1
NC_017661.1
NC_011993.1
NC_022364.1
NC_017644.1
NC_013354.1
NC_013353.1
NC_018650.1
NC_018651.1
NC_018654.1
NC_018652.1
NC_018661.1
NC_018663.1
NC_018662.1
NC_018658.1
NC_018660.1
NC_018666.1
NC_018659.1
NC_013365.1
NC_013370.1
NC_013366.1
NC_013367.1
NC_013368.1
NC_013364.1
NC_011602.1
NC_011603.1
NC_011601.1
NC_007414.1
NC_002655.2
NC_011351.1
NC_011350.1
NC_011353.1
NC_002128.1
NC_002127.1
NC_002695.1
NC_013010.1
NC_013008.1
NC_013369.1
NC_013362.1
NC_013363.1
NC_014543.1
NC_013361.1
NC_013941.1
NC_013942.1
NC_017656.1
NC_017653.1
NC_017657.1
NC_017654.1
NC_017658.1
NC_017655.1
NC_017646.1
NC_017647.1
NC_017648.1
NC_017649.1
NC_017650.1
NC_017634.1
NC_017659.1
NC_017663.1
NC_022370.1
NC_022371.1
NC_011747.1
NC_011742.1

NC_011419.1
NC_011413.1
NC_011416.1
NC_011407.1
NC_011408.1
NC_011411.1
NC_011415.1
NC_013655.1
NC_013654.1
NC_010488.1
NC_010487.1
NC_010486.1
NC_010485.1
NC_010498.1
NC_017652.1
NC_017651.1
NC_010473.1
NC_020518.1
NC_000913.2
AC_000091.1
NC_007779.1
NC_017632.1
NC_017630.1
NC_011749.1
NC_011739.1
NC_011751.1
NC_017641.1
NC_017645.1
NC_017642.1
NC_017640.1
NC_017643.1
NC_017639.1
NC_007941.1
NC_007946.1
NC_017664.1
NC_017635.1
NC_017665.1
NC_017637.1
NC_017662.1
NC_017636.1
NC_017906.1
NC_017907.1
NC_017903.1

Eubacterium limosum
NC_014624.2

Eubacterium rectale
NC_012781.1
NC_021010.1
NC_021044.1

Filifactor alocis
NC_016630.1

Finegoldia magna
NC_010371.1
NC_010376.1

Francisella tularensis
NC_019537.1
NC_019551.1
NC_009749.1
NC_008369.1
NC_007880.1
NC_010677.1
NC_008601.1
NC_008245.1
NC_017453.1
NC_006570.2
NC_009257.1
NC_016937.1
NC_016933.1

Fusobacterium nucleatum
NC_003454.1
NC_022196.1

Gardnerella vaginalis
NC_013721.1
NC_014644.1
NC_017456.1

Gordonia bronchialis
NC_013442.1
NC_013441.1

Haemophilus ducreyi
NC_002940.2

Haemophilus influenzae
NC_016809.1
NC_007146.2
NC_014920.1
NC_014922.1
NC_022356.1
NC_009566.1
NC_009567.1
NC_017452.1
NC_017451.1
NC_000907.1

Haemophilus parainfluenzae
NC_015964.1

Helicobacter cinaedi
NC_020555.1
NC_017762.1
NC_017761.1

Helicobacter pylori
NC_017374.1
NC_017381.1
NC_018939.1
NC_000915.1
NC_017360.1
NC_017382.1
NC_017354.1
NC_017375.1
NC_017357.1
NC_019560.1
NC_019561.1
NC_019562.1
NC_019563.1
NC_019564.1
NC_019565.1
NC_012973.1
NC_014256.1
NC_017358.1
NC_017063.1
NC_017064.1
NC_017368.1
NC_017369.1
NC_017365.1
NC_017370.1
NC_017366.1
NC_017367.1
NC_011334.1
NC_011333.1
NC_008087.1
NC_008086.1
NC_017733.1
NC_017734.1
NC_017372.1
NC_000921.1
NC_017362.1
NC_017363.1
NC_020508.1
NC_020509.1

NC_020556.1
NC_011499.1
NC_011498.1
NC_017742.1
NC_014555.1
NC_014556.1
NC_014257.1
NC_017378.1
NC_017377.1
NC_017379.1
NC_018937.1
NC_018938.1
NC_017359.1
NC_017356.1
NC_017741.1
NC_017740.1
NC_017739.1
NC_010698.2
NC_014560.1
NC_017376.1
NC_017380.1
NC_022130.1
NC_021215.2
NC_021217.2
NC_021218.2
NC_021882.1
NC_021216.2
NC_017355.1
NC_017383.1
NC_017926.1
NC_017919.1

Klebsiella oxytoca
NC_018106.1
NC_021501.1
NC_018107.1
NC_016612.1

Klebsiella pneumoniae
NC_011282.1
NC_011281.1
NC_011283.1
NC_022078.1
NC_022083.1
NC_022082.1
NC_017540.1
NC_017541.1
NC_006625.1
NC_012731.1
NC_018522.1

NC_016845.1
NC_016838.1
NC_016846.1
NC_016839.1
NC_016840.1
NC_016847.1
NC_016841.1
NC_009649.1
NC_009650.1
NC_009651.1
NC_009652.1
NC_009653.1
NC_009648.1
NC_021232.1
NC_021231.1

Legionella longbeachae
NC_014544.1
NC_013861.1

Legionella pneumophila
NC_014125.1
NC_009494.2
NC_006366.1
NC_006369.1
NC_006365.1
NC_006368.1
NC_016811.1
NC_018141.1
NC_020522.1
NC_002942.5
NC_020521.1
NC_021350.1
NC_018139.1
NC_018140.1

Leptospira borgpetersenii
NC_008510.1
NC_008511.1
NC_008508.1
NC_008509.1

Leptospira interrogans
NC_005823.1
NC_005824.1
NC_004342.2
NC_004343.2
NC_017551.1
NC_017552.1

Leptotrichia buccalis
NC_013192.1

Listeria ivanovii
NC_016011.1

Listeria monocytogenes
NC_017728.1
NC_013767.1
NC_013766.1
NC_013768.1
NC_017544.1
NC_018584.1
NC_012488.1
NC_003210.1
NC_017547.1
NC_017546.1
NC_011660.1
NC_017545.1
NC_021830.1
NC_021829.1
NC_018642.1
NC_017529.1
NC_020557.1
NC_017537.1
NC_020558.1
NC_019556.1
NC_018888.1
NC_018591.1
NC_018889.1
NC_018588.1
NC_018590.1
NC_018585.1
NC_018589.1
NC_018586.1
NC_014495.1
NC_018587.1
NC_018592.1
NC_018593.1
NC_002973.6
NC_021823.1
NC_022046.1
NC_021839.1
NC_022047.1
NC_021827.1
NC_022051.1
NC_021840.1
NC_021837.1
NC_021824.1
NC_021825.1

NC_022045.1
NC_021826.1
NC_021828.1
NC_021838.1

Listeria seeligeri
NC_013891.1

Listeria welshimeri
NC_008555.1

Lysinibacillus sphaericus
NC_010381.1
NC_010382.1

Moraxella catarrhalis
NC_014147.1

Mycobacterium abscessus
NC_010397.1
NC_010394.1
NC_021278.1
NC_021279.1
NC_021282.1

Mycobacterium africanum
NC_015758.1

Mycobacterium avium
NC_008595.1
NC_002944.2
NC_021200.1

Mycobacterium bovis
NC_002945.3
NC_020245.2
NC_016804.1
NC_008769.1
NC_012207.1

Mycobacterium leprae
NC_011896.1
NC_002677.1

Mycobacterium marinum
NC_010604.1
NC_010612.1

Mycobacterium smegmatis
NC_019957.1
NC_019958.1
NC_019959.1
NC_019966.1
NC_018289.1
NC_008596.1

Mycobacterium tuberculosis
NC_020089.1
NC_021193.1
NC_017523.1
NC_021251.1
NC_017522.1
NC_002755.2
NC_017524.1
NC_021740.1
NC_021194.1
NC_009565.1
NC_009525.1
NC_018143.1
NC_000962.3
NC_012943.1
NC_016768.1
NC_018078.1
NC_017026.1
NC_017528.1
NC_021054.1
NC_020559.1
NC_022350.1
NC_021192.1
NC_016934.1

Mycobacterium ulcerans
NC_005916.1
NC_008611.1

Mycoplasma fermentans
NC_014552.1
NC_014921.1
NC_021002.1

Mycoplasma genitalium
NC_000908.2
NC_018498.1
NC_018495.1
NC_018496.1
NC_018497.1

Mycoplasma hominis
NC_013511.1

Mycoplasma pneumoniae
NC_016807.1
NC_017504.1
NC_000912.1
NC_020076.1

Neisseria gonorrhoeae
NC_002946.2
NC_011034.1
NC_011035.1
NC_017511.1
NC_017510.1

Neisseria lactamica
NC_014752.1

Neisseria meningitidis
NC_010120.1
NC_017501.1
NC_013016.1
NC_017505.1
NC_008767.1
NC_017513.1
NC_017516.1
NC_017514.1
NC_017517.1
NC_017515.1
NC_003112.2
NC_017518.1
NC_017512.1
NC_003116.1

Nocardia farcinica
NC_006362.1
NC_006363.1
NC_006361.1

Ochrobactrum anthropi
NC_009667.1
NC_009668.1
NC_009669.1
NC_009670.1
NC_009671.1
NC_009672.1

Orientia tsutsugamushi

NC_009488.1
NC_010793.1

Pasteurella multocida
NC_016808.1
NC_017764.1
NC_017027.1
NC_017035.1
NC_002663.1

Porphyromonas asaccharolytica
NC_015501.1

Porphyromonas gingivalis
NC_010729.1
NC_015571.1
NC_002950.2

Prevotella denticola
NC_015311.1

Prevotella intermedia
NC_017860.1
NC_017861.1

Prevotella melaninogenica
NC_014370.1
NC_014371.1

Prevotella ruminicola
NC_014033.1

Propionibacterium acnes
NC_017534.1
NC_017535.1
NC_017550.1
NC_018707.1
NC_021086.1
NC_021085.1
NC_006085.1
NC_014039.1
NC_016512.1
NC_016511.1
NC_016516.1

Proteus mirabilis
NC_022000.1
NC_010554.1
NC_010555.1

Providencia stuartii
NC_017731.1

Pseudomonas aeruginosa
NC_020912.1
NC_018080.1
NC_011770.1
NC_017548.1
NC_017549.1
NC_009656.1
NC_002516.2
NC_021577.1
NC_008463.1

Pseudomonas fluorescens
NC_017911.1
NC_021361.1
NC_016830.1
NC_004129.6
NC_007492.2
NC_009444.1
NC_012660.1

Pseudomonas putida
NC_017530.1
NC_018220.1
NC_009512.1
NC_010322.1
NC_021491.1
NC_019906.1
NC_019905.1
NC_002947.3
NC_021505.1
NC_017986.1
NC_018746.1
NC_015733.1
NC_010501.1

Pseudomonas stutzeri
NC_009434.1
NC_015740.1
NC_018028.1
NC_018177.1
NC_017532.1
NC_019936.1
NC_019937.1
NC_019938.1
NC_019939.1

Rahnella aquatilis
NC_016818.1
NC_016835.1
NC_016819.1
NC_017092.1
NC_017047.1
NC_017060.1
NC_017773.1
NC_017807.1

Ralstonia pickettii
NC_012856.1
NC_012857.1
NC_012855.1
NC_012849.1
NC_012851.1
NC_010682.1
NC_010678.1
NC_010683.1

Rhodococcus equi
NC_014659.1

Rhodococcus erythropolis
NC_022125.1
NC_022115.1
NC_007486.1
NC_007487.1
NC_007491.1
NC_012490.1

Rickettsia africae
NC_012634.1
NC_012633.1

Rickettsia akari
NC_009881.1

Rickettsia australis
NC_017058.1
NC_017041.1

Rickettsia conorii
NC_003103.1

Rickettsia felis
NC_007110.1
NC_007111.1
NC_007109.1

Rickettsia japonica
NC_016050.1

Rickettsia massiliae
NC_009897.1
NC_009900.1
NC_016931.1
NC_016939.1

Rickettsia prowazekii
NC_017560.1
NC_020993.1
NC_017056.1
NC_017049.1
NC_017051.1
NC_017048.1
NC_017050.1
NC_000963.1
NC_020992.1
NC_017057.1

Rickettsia rickettsii
NC_009882.1
NC_016909.1
NC_016913.1
NC_016908.1
NC_016911.1
NC_016914.1
NC_016910.1
NC_016915.1
NC_010263.2

Rickettsia typhi
NC_017062.1
NC_017066.1
NC_006142.1

Rothia dentocariosa
NC_014643.1

Saccharomonospora viridis
NC_013159.1

Salmonella bongori
NC_015761.1
NC_021871.1
NC_021871.1

Salmonella typhi
NC_003384.1
NC_003385.1
NC_003198.1
NC_016825.1
NC_016832.1
NC_004631.1
NC_021176.1

Salmonella typhimurium
NC_003197.1
NC_003277.1

Sebaldella termitidis
NC_013518.1
NC_013519.1
NC_013517.1

Serratia proteamaculans
NC_009829.1
NC_009832.1

Shigella boydii
NC_010656.1
NC_010660.1
NC_010657.1
NC_010659.1
NC_010672.1
NC_010658.1
NC_007608.1
NC_007613.1

Shigella dysenteriae
NC_009344.1
NC_007607.1
NC_007606.1

Shigella flexneri
NC_017328.1
NC_017319.1
NC_017320.1
NC_017329.1
NC_017321.1
NC_017330.1
NC_004741.1
NC_004337.2
NC_004851.1
NC_008258.1

Shigella sonnei
NC_016822.1
NC_016833.1
NC_016823.1
NC_016824.1
NC_016834.1
NC_009345.1
NC_009346.1
NC_009347.1
NC_007385.1
NC_007384.1

Staphylococcus aureus
NC_017340.1
NC_018608.1
NC_021657.1
NC_021670.1
NC_021552.1
NC_021554.1
NC_021059.1
NC_021060.1
NC_007622.1
NC_017351.1
NC_017350.1
NC_022126.1
NC_022113.1
NC_022222.1
NC_017673.1
NC_022227.1
NC_022228.1
NC_022226.1
NC_006629.2
NC_002951.2
NC_017346.1
NC_017344.1
NC_017343.1
NC_017337.1
NC_013453.1
NC_013451.1
NC_013452.1
NC_013450.1
NC_017763.1
NC_009619.1
NC_009632.1
NC_009477.1
NC_009487.1
NC_017338.1
NC_017339.1
NC_017348.1
NC_017349.1
NC_016928.1

NC_002952.2
NC_016941.1
NC_005951.1
NC_002953.3
NC_009782.1
NC_002774.1
NC_002758.2
NC_003923.1
NC_003140.1
NC_002745.2
NC_007795.1
NC_016942.1
NC_017333.1
NC_022443.1
NC_022442.1
NC_020529.1
NC_020564.1
NC_020532.1
NC_020533.1
NC_020537.1
NC_020566.1
NC_020536.1
NC_020568.1
NC_020530.1
NC_020531.1
NC_020565.1
NC_020534.1
NC_020535.1
NC_020567.1
NC_020538.1
NC_020539.1
NC_017334.1
NC_017335.1
NC_017336.1
NC_017341.1
NC_009641.1
NC_017347.1
NC_017342.1
NC_017345.1
NC_017352.1
NC_017332.1
NC_017331.1
NC_007790.1
NC_007791.1
NC_007792.1
NC_007793.1
NC_010063.1
NC_012417.1
NC_010079.1
NC_016912.1

Staphylococcus epidermidis
NC_005008.1
NC_005007.1
NC_005006.1
NC_005005.1
NC_005004.1
NC_005003.1
NC_004461.1
NC_006663.1
NC_002976.3

Staphylococcus haemolyticus
NC_007169.1
NC_007170.1
NC_007171.1
NC_007168.1

Staphylococcus lugdunensis
NC_013893.1
NC_017353.1

Staphylococcus saprophyticus
NC_007351.1
NC_007352.1
NC_007350.1

Stenotrophomonas maltophilia
NC_017671.1
NC_010943.1
NC_011071.1

Streptobacillus moniliformis
NC_013516.1
NC_013515.1

Streptococcus agalactiae
NC_021485.1
NC_021195.1
NC_004116.1
NC_007432.1
NC_018646.1
NC_021486.1
NC_021507.1

NC_004368.1
NC_019048.1

Streptococcus equi
NC_012471.1
NC_017582.1
NC_011134.1
NC_012470.1

Streptococcus gordonii
NC_009785.1

Streptococcus intermedius
NC_022246.1
NC_022237.1
NC_018073.1

Streptococcus mitis
NC_013853.1

Streptococcus mutans
NC_018089.1
NC_017768.1
NC_013928.1
NC_004350.2

Streptococcus pneumoniae
NC_014498.1
NC_012468.1
NC_014494.1
NC_011900.1
NC_010582.1
NC_008533.1
NC_011072.1
NC_018630.1
NC_010380.1
NC_017591.1
NC_017593.1
NC_012466.1
NC_017592.1
NC_012467.1
NC_003098.1
NC_021006.1
NC_021028.1
NC_021026.1
NC_021005.1
NC_018594.1
NC_017769.1
NC_012469.1

NC_014251.1
NC_003028.3

Streptococcus pyogenes
NC_018936.1
NC_017596.1
NC_021807.1
NC_020540.2
NC_002737.1
NC_008022.1
NC_006086.1
NC_008024.1
NC_017040.1
NC_017053.1
NC_008023.1
NC_004070.1
NC_007297.1
NC_007296.1
NC_003485.1
NC_008021.1
NC_011375.1
NC_004606.1
NC_009332.1

Streptococcus salivarius
NC_017594.1
NC_015760.1
NC_017595.1

Streptococcus suis
NC_009442.1
NC_009443.1
NC_017622.1
NC_012923.1
NC_012926.1
NC_017621.1
NC_017620.1
NC_017617.1
NC_017618.1
NC_012925.1
NC_018526.1
NC_020526.1
NC_012924.1
NC_017619.1
NC_017950.1
NC_015433.1
NC_021213.1

Streptococcus uberis
NC_012004.1

Treponema pallidum
NC_021179.1
NC_016844.1
NC_010741.1
NC_021508.1
NC_017268.1
NC_018722.1
NC_000919.1
NC_021490.2
NC_016848.1
NC_016843.1
NC_016842.1

Tsukamurella paurometabola
NC_014158.1
NC_014159.1

Ureaplasma urealyticum
NC_011374.1

Veillonella parvula
NC_013520.1

Vibrio cholerae
NC_016944.1
NC_016945.1
NC_017270.1
NC_017269.1
NC_012578.1
NC_012580.1
NC_012668.1
NC_012667.1
NC_002505.1
NC_002506.1
NC_016445.1
NC_016446.1
NC_009456.1
NC_009457.1
NC_012582.1
NC_012583.1

Vibrio furnissii
NC_016602.1
NC_016628.1

Vibrio parahaemolyticus
NC_019955.1
NC_019971.1
NC_021848.1
NC_021822.1
NC_021847.1
NC_021821.1
NC_004603.1
NC_004605.1

Vibrio vulnificus
NC_004459.3
NC_004460.2
NC_014965.1
NC_014966.1
NC_005139.1
NC_005140.1
NC_005128.1

Wolinella succinogenes
NC_005090.1

Yersinia enterocolitica
NC_008791.1
NC_008800.1
NC_015224.1
NC_015475.1
NC_017565.1
NC_017564.1

Yersinia pestis
NC_017168.1
NC_017169.1
NC_017170.1
NC_010157.1
NC_010158.1
NC_010159.1
NC_008122.1
NC_008120.1
NC_008121.1
NC_008150.1
NC_017265.1
NC_017263.1
NC_017266.1
NC_017264.1
NC_005813.1
NC_005814.1
NC_005815.1
NC_005816.1
NC_005810.1
NC_003131.1
NC_003134.1
NC_003132.1

NC_003143.1
NC_017154.1
NC_017153.1
NC_017155.1
NC_017156.1
NC_017160.1
NC_017157.1
NC_017158.1
NC_017159.1
NC_004088.1
NC_004838.1
NC_008118.1
NC_008119.1
NC_008149.1
NC_009377.1
NC_009378.1
NC_009381.1
NC_014029.1
NC_014017.1
NC_014022.1
NC_014027.1

Yersinia
pseudotuberculosis
NC_009705.1
NC_009704.1
NC_009708.1
NC_006154.1
NC_006153.2
NC_006155.1
NC_010635.1
NC_010634.1
NC_010465.1
